# Immediate effect of different exercises in the vocal space of women with and without vocal nodules

**DOI:** 10.1590/2317-1782/20212021157en

**Published:** 2022-07-22

**Authors:** Fernanda Pereira França, Anna Alice Almeida, Leonardo Wanderley Lopes

**Affiliations:** 1 Universidade Federal da Paraíba – UFPB, João Pessoa, PB, Brasil.

**Keywords:** Voice, Voice Disorders, Acoustic, Laryngeal Diseases, Voice Training

## Abstract

**Purpose:**

To investigate the immediate effect of voiced tongue vibration (VSL), high-resistance straw in the air (CAR), and overarticulation (OA) on the vocal space of vocally healthy women (MVS) and with vocal nodules (MNV).

**Methods:**

12 women participated in the MNV and 12 women in the MVS, allocated to perform the vocal exercises of VSL, CAR, and OA. Each participant performed only one of the three proposed exercises, for 5 minutes, preceded and followed by recording a sequence of vehicle sentences for extracting formants (F1 and F2) from the vowel segments [a, i, u]. The vowel space was analyzed through the differences between the measures of the formants of the vowels.

**Results:**

we observed a reduction of F1 in the interval [a]-[i] and [i]-[u] and of F2 between the vowels [a]-[u] and [i]-[u] in the MVS, after performing the CAR. In MNV, we observed a reduction of F2 in the interval [a]-[i] after VSL. In the intergroup analysis, there were higher F1 values between the intervals of the vowels [a]-[i] and [i]-[u] in the MVS, before performing the CAR, and after exercise only in the interval [a]-[i]. A higher value of F1 and F2 was observed in the interval between the vowels [i]-[u] in the MNV after VSL.

**Conclusion:**

The VSL exercise reduced the vowel space in MNV women. CAR reduced the vocal space of women in the MVS. The MNV had a smaller vowel space compared to the MVS before and after the CAR. We observed a reduction in the vowel space in the MNV compared to the MNV after the VSL exercise.

## INTRODUCTION

Formant measures are the main acoustic correlates associated with the description of vowel segments. Formants are the result of air pulses that pass through the vocal folds, causing the walls of the vocal tract to vibrate, producing energy concentration zones^(1)^.

From the formant analysis, it is possible to investigate the movement and positioning of the articulators (jaw, lips, and tongue) during speech. In this sense, different combinations in the positioning of these articulators contribute to the acoustic-articulatory distinctiveness of the vowel segments^(2)^.

The vowels [a, i, u] are the ones that are most acoustically distinguished since they are found at the ends of the articulatory triangle1. In general, the language is in a higher position in the production of the vowels [i] and [u], which contributes to the lower values of the frequency of the first formant (F1) for these vowels. In turn, the oromandibular complex tends to be lower in the production of the vowel [a], which can increase the frequency of F11. The second formant (F2) has higher frequency values in the vowel [i], justified by the more anterior position of the tongue. The frequency of F2 tends to be lower in the vowel [u], due to the posterior displacement of the tongue, and assumes intermediate values between the vowels [i] and [u] in the production of the vowel [a]^(1,3)^.

The triangle formed by the frequencies of the vowel formants can then be represented by a graphic method through a diagram that joins two coordinates, according to the value of the first and second formants, with F2 on the abscissa and F1 on the ordinate. The area created inside the triangle is called vowel space, based on the Euclidean distance between the vowels [i] – [a] – [u]. An enlarged space of this triangle can be one of the markers of greater acoustic distinctiveness between the vowels and, consequently, a greater range of movement of the articulators, while the reduction of the vowel space can indicate the opposite. In addition, vowel distinctiveness is one of the factors responsible for speech intelligibility, specifically with regard to vowels in a given language^(3)^.

Traditionally, formant measures have been used for different purposes, which include the characterization of vowel space in different populations as a function of gender, age, linguistic variation, speech styles and individuals with communication disorders of different etiologies. For example, vowel space has been used to assess the relationship between vowel articulation and speech intelligibility in individuals with Parkinson's disease, stroke sequelae, children with Down syndrome, with cleft palate, among others^(4-6)^. Thus, dysarthric patients present a reduction in the vowel space due to the decrease in the amplitude and speed of the movements of the articulators. One of the main effects of the treatment in these patients is the expansion of the vowel space^(7)^.

The presence of a voice disorder can also interfere with speech intelligibility and the vowel space area. Dysphonic individuals may have lower speech intelligibility^(8)^, reduced vowel space^(9)^, with lower values of F1 and F2 for the oral vowels [a], [i] and [u] of Brazilian Portuguese in relation to vocally healthy women^(10)^. Furthermore, an enlargement of the vowel space was observed in patients with dysphonia due to primary muscle tension after treatment with manual circumlaryngeal therapy^(11)^.

Thus, it is known that the modification of laryngeal activity due to the presence of a functional or structural alteration can result in dysfunctional supraglottic adjustments (such as supraglottic constriction, reduction of the oropharyngeal space and elevation-anteriorization of the hyolaryngeal complex) that co-occur or are a consequence of such amendments^(10,12)^. In this context, vocal exercises are used in the vocal rehabilitation process for possible modification of dysfunctional adjustments, causing effects with different focuses, either in the glottic source, in the vocal tract or the interaction between the glottic source and the vocal tract^(13,14)^.

The selection of exercises in the vocal clinic should therefore be directed according to the main complaint or needs of the patient, his/her vocal behavior, and his/her vocal demand. The main objectives of vocal exercises include rebalancing the subsystems involved in vocal production; improving balance, tone and resistance of the muscles involved in vocal production; promoting the development of healthy mucoondulatory movement of the vocal folds and reinforcing new behaviors related to voice use^(15)^.

However, acoustic analysis, using measures of perturbation, energy and noise more strictly related to the glottic source, is the main reference standard used to monitor the immediate effect of clinical intervention^(16)^. Although formant measures have been extensively investigated related to vocal production of vocally healthy individuals or with voice disorders, the main interest was related to the process of identification, characterization of the disorder or diagnostic confirmation/differentiation of vocal and laryngeal disorders.

In recent years, functional magnetic resonance imaging^(13)^ and acoustic pharyngometry^(17)^ have included the investigation of the effect of vocal exercises on the supraglottic region. It should be considered that these technologies mentioned are not accessible for use in clinical routine and that the study of formant measures, specifically, of the vowel space pre- and post-intervention^(11)^, can provide inferences about the effect of vocal exercises on the positioning and movement of articulators before and after vocal exercise. In addition, formant measurements are considered the main reference standard for validating imaging studies related to the vocal tract and for building physical models for the synthesis of human voices^(18)^.

Therefore, the research question that motivated this study can be defined as follows: does the primary focus of action of vocal exercises (glottic source, source-filter coupling or range of motion of the articulators) cause different effects on the vowel space of oral vowels [a, i, u] in vocally healthy women with vocal nodules?

In this context, this study aimed to investigate the immediate effect of voiced tongue vibration (VSL), high-resistance straw in the air (CAR) and overarticulation (OA) on the vocalic space of vocally healthy women with vocal nodules.

## METHODS

### Study design

This is an analytical and intervention study. It was evaluated and approved by the Research Ethics Committee of the Institution of origin, with report number 2,158,960. All participants received an explanation about the research and signed an informed consent form.

### Sample

To carry out this research, two groups were constituted: women with vocal nodules (MNV group), with the participation of adult women, assisted at the Voice Laboratory of a Higher Education Institution; and the vocally healthy women group (MVS), formed by adult women without voice disorders. For the MNV, the following eligibility criteria were considered:

- Present an otorhinolaryngological report of vocal nodules;
- Be female, due to the higher prevalence of voice disorders in this gender and the influence of this variable on the values of the mean fundamental frequency (f0) and formant measures, which present differences between adult men and women, due to the anatomical characteristics of the vocal folds and vocal tract;
- Ages 19 and 59 years, to avoid changes in the glottic source and the vocal tract related to childhood, adolescence, and senescence;

For the composition of the group Vocally Healthy Women (MVS), female employees and students of an undergraduate course at the Higher Education Institution where the research was carried out were actively recruited, who were available for the realization of the same and who fit the same criteria. Eligibility criteria cited for the MNV, except for the diagnosis of vocal nodules and:

- Not have a voice complaint at the time of collection or in the previous six months, responding negatively to the question “do you have a voice problem currently or in the last six months?”
- Present an otorhinolaryngological report of a normal larynx;

In both groups, the following exclusion criteria were considered:

- Present upper airway infections at the time of recording, which would generate changes in resonance cavities and, consequently, in formant measurements;
- Have a shortened lingual frenulum, temporomandibular disorders, and/or structural and functional changes in the articulators, which would modify the adjustments of the supraglottic vocal tract;
- Have cognitive or neurological changes that prevent the performance of collection procedures;
- Have undergone speech-language therapy before.

The age range variable was used to match the MVS with the MNV, considering an age variation of more or less five years, following a proportion of one control for each case (1:1).

All MNV women were recruited at the time of the initial consultation at the Laboratory where the research was carried out, before starting the vocal therapy process itself. Initially, the researchers had access to the patients' evaluation form to verify the sociodemographic data (age) and the laryngeal diagnosis made by the otorhinolaryngologist before the initial consultation for vocal evaluation, according to the routine of the aforementioned Laboratory. Those who met the defined eligibility criteria were approached at the end of the vocal assessment session and invited to participate in the research, after reading and agreeing with the informed consent. Thus, the MNV sample consisted of 12 women with a mean age of 36.47 ± 12.22 years.

For the recruitment of women from the MVS, the observation of the age group of the participants in the MNV was considered. Therefore, they were contacted by the researcher, who provided information about the research and, in case of consent of the volunteer and signature of the ICF, information was collected in relation to age and presence of vocal complaint. Those who met the eligibility criteria for the MVS were referred for a laryngeal visual examination (videolaryngoscopy) with an otorhinolaryngologist to rule out the presence of functional or structural alterations in the larynx. After the results of the exams, women with a report of “normal larynx” were recruited to continue their participation in the study. Thus, the MVS sample consisted of 12 women with a mean age of 33.86 ± 11.59 years.

### Data collection procedures

The MNV and MVS participants selected based on the eligibility criteria were invited to participate in a session for voice collection and intervention. At the beginning of the session, the research objectives were resumed after reading the ICF and confirming personal data, including.

Data collection took place in three stages, namely: 1) evaluation of the stomatognathic system structures and initial collection of speech tasks; 2) performance of vocal exercises; 3) and voice collection after vocal exercise.

**Step 1** - Assessment of the stomatognathic system structures and initial collection of speech tasks.

Initially, an evaluation of the structures of the stomatognathic system was performed, observing morphology and mobility of the lips, tongue, cheeks and soft palate; tonicity of lips, tongue and cheeks; mouth opening amplitude and presence of complaints related to the temporomandibular joint; and the presence of complaints of upper airway infections (according to self-report). The objective of this evaluation was to rule out the presence of temporomandibular disorder, alteration in the lingual frenulum, or any structural and functional alteration that could influence the results of this study, due to the interference of these alterations on the articulatory adjustments.

Afterward, the speech tasks were recorded. For that, we used the Fonoview software, version 4.5, from CTS Informática, a Dell all-in-one desktop, unidirectional cardioid microphone, Sennheiser, model E-835, located on a pedestal and coupled to a Behringer preamplifier., model U-Phoria UMC 204. The voices were collected in a recording booth, in a voice studies laboratory, with acoustic treatment and noise lower than 50 dB SPL, verified with a sound pressure level meter R8050 from Reed Instruments; with a sampling rate of 44000 Hz, 16 bits per sample and a distance of 10 cm between the microphone and the speaker's mouth.

To collect the voices, the women stood up, placing the pedestal in front of them, according to the recommended distance between the mouth and the microphone, as described above. They were instructed to take a breath in the usual way and, subsequently, to repeat each of the following vehicle phrases three times, separately: “I say p **a** pa softly”, “I say p **i** pa softly” and “I say p **u** pa softly”.

The quoted sentences contain the vowel segments [a, i, u] in CV context (consonant - vowel), in the initial syllable of a word, unaccented, with a vowel preceded and followed by the voiceless bilabial stop phoneme. The choice of this phrase is justified by the little influence that these consonants have on the formants of adjacent vowels^(3)^ and by the need to homogenize the phonetic context to obtain all the vowels of the speech samples. Thus, we observed minimal control of the prosodic aspects, without interference in the performance of vowel sounds in the investigation of the acoustic distinctiveness of vowels.

The vowels [a, i, u] were chosen due to the recognized acoustic distinctiveness between them, forming a vowel articulatory triangle at their ends^(3)^. In addition, they obey a formant pattern of consensus among researchers, which corresponds to the typical characteristics of vowels that present the maximum and minimum of vowel opening and of movement of retreat and advance, of lowering and lifting of the tongue.

**Step 2** - Performing vocal exercises

After this initial collection of the speech tasks, the informants were oriented about the next experimental procedures of the research, related to the execution of the vocal exercises. For this research, three exercises frequently used in voice therapy and widely cited in the literature^(14,19,20)^ were chosen, including the VSL, the CAR and the OA.

To select these exercises, the following criteria were used:

- Exercises routinely used in vocal therapy and cited in speech-language therapy intervention studies in the rehabilitation of voice disorders^(21,22)^;
- Exercises whose physiological principles of action described in the literature in the area involved defined and, as far as possible, independent effects regarding the glottic source^(19)^, improved source-filter interaction^(14)^ and changes in the movement and positioning of the articulators^(20)^.

According to the taxonomy of approaches used in voice therapy^(23)^, VSL is considered an exercise classified within the vocal function category, characterized as a type of intervention that directs the patient's attention to modifying the phonatory (glottic) adjustments themselves; the CAR exercise classified in the category of somatosensory approach, whose main objective is to direct the patient's attention to the modification of the somatic input; and OA is classified within the musculoskeletal approach, directing the patient's attention to the muscular modification related to the movements of the structures of the oral cavity.

Thus, the three exercises basically focus on the glottic source (VSL), the movement of the vocal tract articulators (OA) and the coupling of the source and the vocal tract (CAR). The study of these techniques will favor the understanding of the interaction between the sound source and the mechanisms of sound articulation, describing their influences on communication, as well as reproducing the physical properties inherent to the production of speech^(10,12)^.

Initially, the three techniques were presented to the participants and asked to perform each one of them once. As we perceived the ease of execution or based on the individual's report regarding the difficulty of execution, we chose the technique that would be performed by this volunteer, advocating the technique produced with greater ease and less effort by the participant. The objective of this procedure was to exclude the possibility that the difficulty in the execution influenced the effect of the technique. If the participant presented these requirements in more than one exercise, the allocation would be made for convenience, to compose the parity of the groups. Thus, the participants of the MNV and the MVS were allocated into three groups (A, B and C), depending on the vocal exercise performed. Each volunteer necessarily participated in only one of the groups.

Group A, composed of four women from the MNV (mean age ±35 years) and four women from the MVS (mean age ±30.75 years), performed the VSL exercise. In this sense, the participants were asked to emit the sound of the phoneme [ɾ] in a sustained manner at a self-reported comfortable frequency and intensity. The choice of this exercise is because it can enable changes in the glottic source, with a direct influence on the physiology of the vocal folds^(19)^. Performing this technique helps in the resorption of benign vocal fold lesions, modifying the hyperkinetic condition found in cases of vocal nodules, resulting in increased blood supply to the vocal folds, reducing elastic and viscous resistance.

Group B, composed of five women from the MNV (mean age ±36 years) and five women from the MVS (mean age ±35.8 years), performed the exercise with CAR. To perform this exercise, a rigid plastic straw, 8.7 cm long and 1.5 mm in diameter, was used. The group members were instructed to emit a sound similar to [vu] in a sustained manner at a self-reported comfortable frequency and intensity. They were instructed that the entire sound flow came out through the straw, as demonstrated by the researcher.

The choice of this exercise was justified by the effect it has on the vocal production system, adjusting the vocal folds and vocal tract, increasing the interaction between the glottic source and the supraglottic vocal tract^(21)^. The vocal folds vibrate more smoothly, because they are slightly separated (abducted) by the action of pressure that closes the air outlet generated inside the “tube”. Airflow is also reduced, as well as the impact of contact between the vocal folds, also helping in the resorption of the mass lesion between the vocal folds^(22)^.

Group C, composed of three women from the MNV (mean age ±34 years) and three women from the MVS (mean age ±34.66), performed the OA exercise. As for the OA exercise, the group members were instructed to exaggerate their articulatory movements, making a greater muscular excursion, with greater mouth opening and greater range of lip movement, while reading a phonetically balanced text, based on the Brazilian Portuguese version of Vocal Profile Analysis Scheme – PB-VPAS (2007).

The choice of OA exercise was based on the physiology found during its performance, since it involves the movement of the articulators, focusing their performance on the vocal tract, but to reduce laryngeal hypertonicity, improve speech articulation and vocal projection.

Subsequently, for each vocal exercise, 5 sets of 1 minute were performed, totaling 5 minutes of execution21, with the vocal recording of the three vehicle phrases described above, before and after the five minutes of execution.

Participants in the MVS group were recruited after collection with the MNV group. This procedure was defined to favor the same number of informants in both groups.

The extraction of the first and second formants of the vowels [a, i, u] was performed using the Praat software, version 5.3.77h, based on the representation of the vowel in a broadband spectrogram. Praat is a voice analysis tool developed by Paul Boersma and David Weenink from the Institute of Phonetic Sciences, University of Amsterdam.

From the selection and segmentation of vowel sounds in CV contexts, it was possible to obtain the average of the investigated acoustic measures. For the extraction of the average of the formants in Praat, the option called Formant was selected, obtaining the numerical value of F1, F2 and F3 expressed in Hertz (Hz).

### Data analysis procedure

For data analysis and discussion, the performance of the exercise was considered as follows: m0 – before performing the vocal exercise; m5 – after the fifth minute of vocal exercise.

Statistical analysis was performed considering descriptive measures, such as mean and standard deviation of differences between vowels (vowel space), that is, the result of subtracting the means of vowels [a]-[u]; [a]-[i] and [i]-[u], concerning the first and second formants.

For intragroup comparisons (MNV and MVS) between the intervals of the spaces of the vowels [a]-[u], [a]-[i] and [i]-[u] of F1 and F2, the t-tests of Paired Student and Wilcoxon for paired data. When the normality assumption was not satisfied, the t-test was replaced by Wilcoxon's non-parametric test. In the intergroup comparison (MVS vs. MNV) Student's t-test and Wilcoxon's nonparametric test were used, considering independent samples.

All analyzes were performed using the R software. The significance level considered was 5%.

## RESULTS

Table 1 shows the averages and standard deviation of the differences between F1 and F2 of the vowels, between the intervals [a]-[u], [a]-[i] and [i]-[u] in the moments m0 and m5 of all vocal techniques investigated. This also exposes data from the intragroup comparative analysis, based on the means of the vowel differences of F1 and F2, according to each vowel interval analyzed, before and after performing the vocal exercises.

**Table 1:** Mean and standard deviation of vowel interval differences and intragroup comparison between vowel F1 and F2 differences at pre and post 5 minutes

**Exercise**		**MNV**								
		**[a] – [u]**			**[a] – [i]**			**[i] – [u]**		
		**Time m0**	**Time m5**	**p-value**	**Time m0**	**Time m5**	**p-value**	**Time m0**	**Time m5**	**p-value**
		**Mean SD**	**Mean SD**		**Mean SD**	**Mean SD**		**Mean SD**	**Mean SD**	
Tongue Vibration	F1	533.08±182.57	397.74±107.21	0.1869	500.45±150.72	416.15±120.90	0.1083	32.63±103.93	18.40±17.98	0.1083
	F2	682.44±204.27	654.2±121.80	0.775	1153.32±132.90	1021.81±190.52	0.0274	1835.76±322.39	1676.01±253.65	0.0857
High Strength Straw	F1	350.05±154.38	344±109.92	0.8533	375.24±150.75	372.11±98.29	0.9303	25.19±8.41	28.11±29.56	0.8386
	F2	562.93±296.95	647.10±198.89	0.5448	1078.63±279.35	1027.80±287.64	0.4509	1641.56±425.82	1674.90±291.13	0.693
Overarticulation	F1	447.34±86.17	458.8±39.26	0.8222	512.62±114.15	560.35±62.24	0.2912	65.28±42.87	101.55±79.67	0.2348
	F2	761.10±89.08	634.66±203.23	0.531	1120.59±64.25	1206.87±130.93	0.1675	1881.69±45.97	1841.50±333.48	0.8652
		**MVS**								
		**[a] – [u]**			**[a] – [i]**			**[i] – [u]**		
**Exercise**		**Time m0**	**Time m5**	**p-value**	**Time m0**	**Time m5**	**p-value**	**Time m0**	**Time m5**	**p-value**
		**Mean SD**	**Mean SD**		**Mean SD**	**Mean SD**		**Mean SD**	**Mean SD**	
Tongue Vibration	F1	433.07±33.53	385.18±85.89	0.3776	503.39±40.66	475.74±116.76	0.6224	70.32±38.02	90.56±44.27	0.4355
	F2	680.42±94.69	631.73±47.87	0.3709	1002.12±91.60	1042.42±61.66	0.1681	1682.55±165.62	1674.15±75.07	0.9081
High Strength Straw	F1	491.57±59.06	443.42±40.80	0.1578	601.85±68.16	513.53±73.98	0.0251	110.27±69.67	70.10±50.33	0.0498
	F2	755.13±78.37	697.72±72.27	0.0463	1195.64±100.49	1173.79±26.60	0.6138	1950.78±100.21	1871.52±57.68	0.0419
Overarticulation	F1	480.15±98.72	496.08±84.37	0.7019	541.55±110.48	552.67±87.58	0.8038	61.40±18.13	56.59±13.08	0.302
	**F2**	815.17±115.34	772.93±70.67	0.2833	1098.24±90.67	1135.99±70	0.2833	1913.41±47.31	1908.92±127.90	0.9424

Significant values (p<0.05) Paired t test; Paired Wilcoxon

Caption: MNV = female group with vocal nodules; MVS = vocally healthy women group; SD = standard deviation; F1= first formant; F2= second formant; m: moment

**Figure 1:**
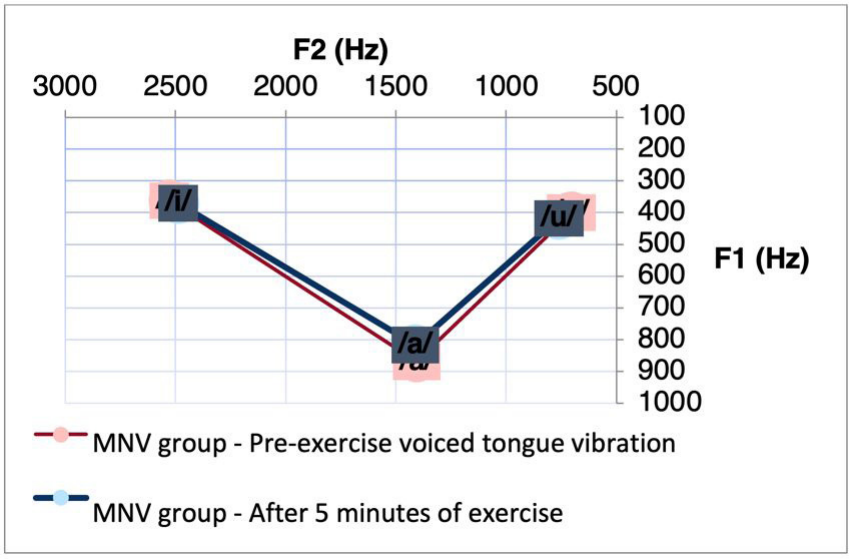
Acoustic-articulatory triangle of the vowels [a], [i] and [u] of the MNV group before and after 5 minutes of the VSL exercise

The MNV group presented a significant difference for the F2 after performing the VSL exercise in the intervals of the vowels [a]-[i] (p = 0.0274) (Table 1). We observed a reduction in the intervals of these vowels after the exercise. Figure 1 shows the decrease in vowel space between these vowels in the condition specified for the MNV.

**Figure 2:**
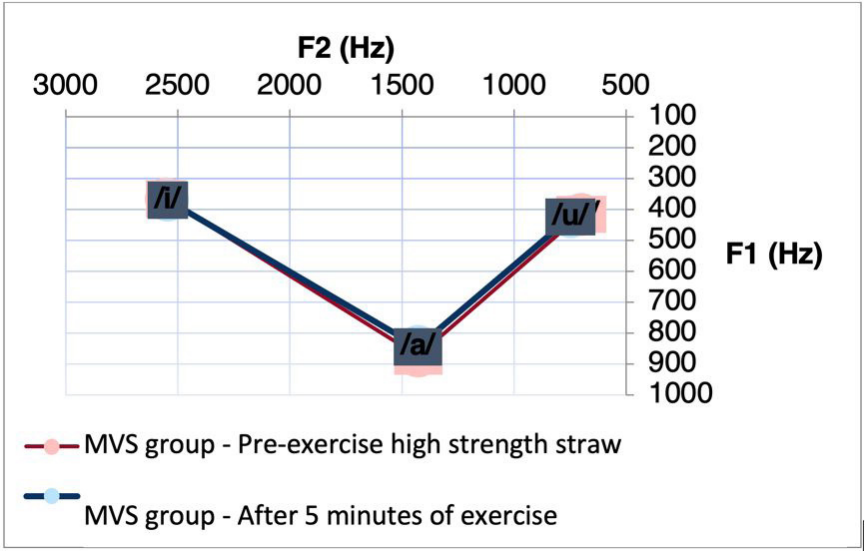
Acoustic-articulatory triangle of the vowels [a], [i] and [u] of the MVS group before and after 5 minutes of the exercise with CAR

Regarding the MVS group, after performing the CAR exercise, we observed a significant difference for F1 between the vowel intervals [a]-[i] (p= 0.0251) and [i]-[u] (p = 0, 0498), and for the F2 between the vowel intervals [a]-[u] (p = 0.0463) and [i]-[u] (p = 0.0419) (table 1). We observed a reduction in the intervals of these vowels after the exercise. Figure 2 shows the decrease in vowel space between these vowels in the condition specified for the MVS.

**Figure 3:**
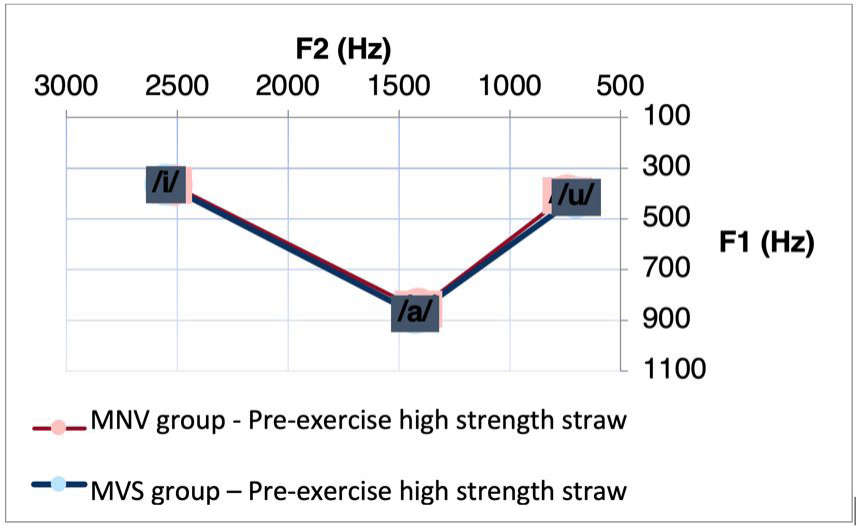
Acoustic-articulatory triangle of the vowels /a/, /i/ and /u/ of the group of individuals with nodules and control before performing the exercise with CAR

**Figure 4:**
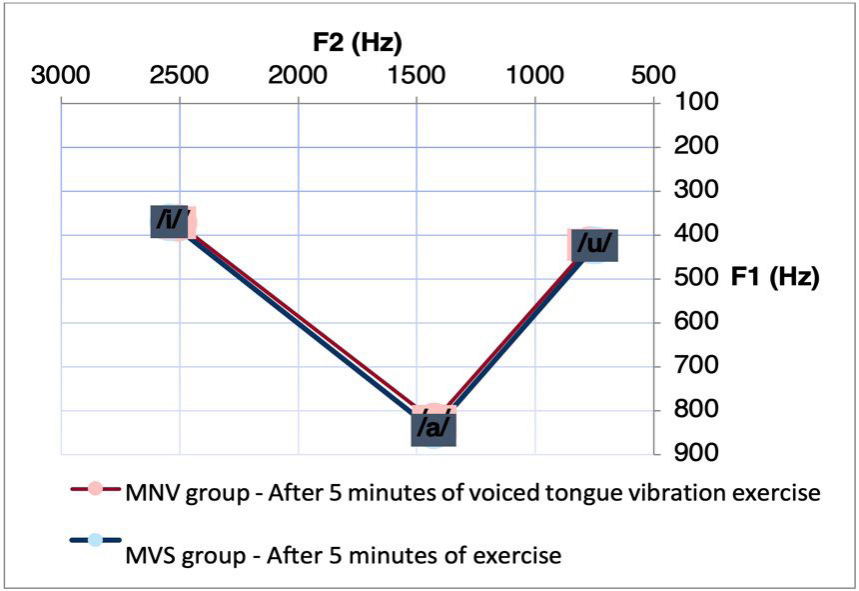
Acoustic-articulatory triangle of the vowels [a], [i] and [u] of the MNV and MVS group after 5 min. of carrying out the VSL exercise

**Figure 5:**
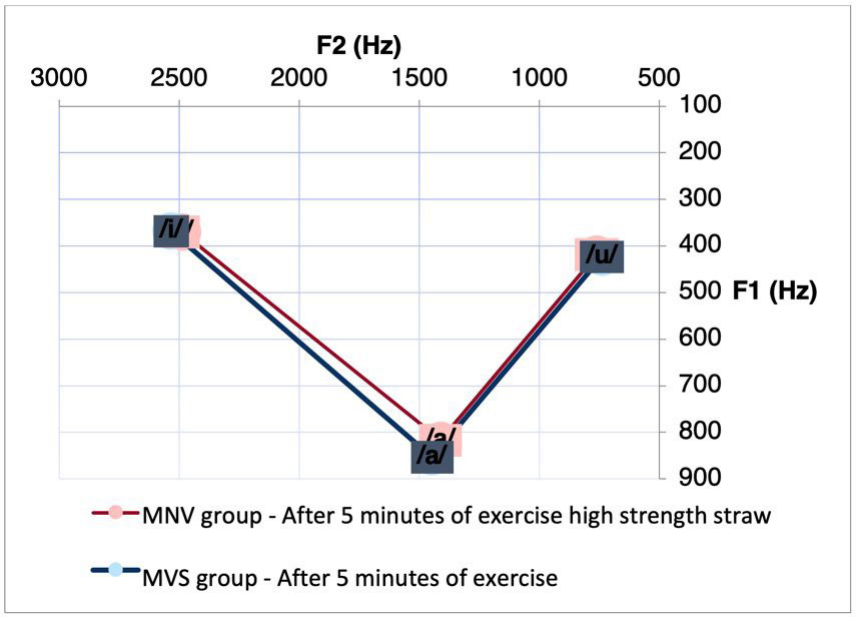
Acoustic-articulatory triangle of vowels [a], [i] and [u] of the MNV and MVS group after 5 min. of carrying out the exercise with CAR

Next, Table 2 shows a comparison analysis between the MNV and MVS regarding the vowel interval based on F1 and F2, at moments m0 and m5 of all vocal techniques investigated. Before performing the exercises, we observed a difference for F1 between the interval [a]-[i] (p = 0.02436) and [i]-[u] (p = 0.04999) in the CAR group. It is possible to observe smaller values ​​between these intervals in the MNV group. Figure 3 shows the decrease in vowel space in this group. After performing the VSL exercise, we observed a difference between the groups for F1 (p = 0.0396) and F2 (p = 0.0285) between the interval [i]-[u]. And after performing the CAR exercise, we observed a difference between the groups for F1, in relation to the interval [a]-[i] (p = 0.03517). It is observed that after performing the VSL and CAR, women with vocal nodules, from both groups, present a reduction in the values ​​in these mentioned intervals. Figures 4 and 5 show the decrease in vowel space in the MNV in the condition specified for the group that performed the VSL and CAR, respectively.

**Table 2:** Mean and standard deviation of vowel interval differences and intergroup comparison between vowel F1 and F2 differences at pre and post 5 minutes

**Exercise**		**Time m0**								
		**[a] – [u]**			**[a] – [i]**			**[i] – [u]**		
		**MNV**	**MVS**	**p-value**	**MNV**	**MVS**	**p-value**	**MNV**	**MVS**	**p-value**
		**Mean SD**	**Mean SD**		**Mean SD**	**Mean SD**		**Mean SD**	**Mean SD**	
Tongue Vibration	F1	533.08±182.57	433.07±33.53	0.3556	500.45±150.72	503.39±40.66	0.9720	32.63±103.93	70.32±38.02	0.1403
	F2	682.44±204.27	680.42±94.69	0.9865	1153.32±132.90	1002.12±91.60	0.1163	1835.76±322.39	1682.55±165.62	0.4372
High Strength Straw	F1	350.05±154.38	491.57±59.06	0.1121	375.24±150.75	601.85±68.16	0.0243	25.19±8.41	110.27±69.67	0.0499
	F2	562.93±296.95	755.13±78.37	0.2260	1078.63±279.35	1195.64±100.49	0.4184	1641.56±425.82	1950.78±100.21	0.1821
Overarticulation	F1	447.34±86.17	480.15±98.72	0.6873	512.62±114.15	541.55±110.48	0.7683	65.28±42.87	61.40±18.13	0.8953
	F2	761.10±89.08	815.17±115.34	0.5576	1120.59±64.25	1098.24±90.67	0.7469	1881.69±45.97	1913.41±47.31	0.4519
		**Tempo m5**								
		**[a] – [u]**			**[a] – [i]**			**[i] – [u]**		
**Exercise**		**MNV**	**MVS**	**p-value**	**MNV**	**MVS**	**p-value**	**MNV**	**MVS**	**p-value**
		**Mean SD**	**Mean SD**		**Mean SD**	**Mean SD**		**Mean SD**	**Mean SD**	
Tongue Vibration	F1	397.74±107.21	385.18±85.89	0.8612	416.15±120.90	475.74±116.76	0.5049	18.40±17.98	90.56±44.27	0.0396
	F2	654.2±121.80	631.73±47.87	0.7490	1021.81±190.52	1042.42±61.66	0.8479	1727.2±253.65	1794.5±75.07	0.0285
High Strength Straw	F1	344±109.92	443.42±40.80	0.1155	372.11±98.29	513.53±73.98	0.0351	28.11±29.56	70.10±50.33	0.1553
	F2	647.10±198.89	697.72±72.27	0.6155	1027.80±287.64	1173.79±26.60	0.5476	1674.90±291.13	1871.52±57.68	0.2076
Overarticulation	F1	458.8±39.26	496.08±84.37	0.5404	560.35±62.24	552.67±87.58	0.9081	101.55±79.67	56.59±13.08	0.7000
	F2	634.66±203.23	772.93±70.67	0.4000	1206.87±130.93	1135.99±70	0.3619	1841.50±333.48	1908.92±127.90	0.7684

Significant values (p<0.05) Paired t test; Paired Wilcoxon

Caption: MNV = group of women with vocal nodules; MVS = vocally healthy women group; SD = standard deviation; F1= first formant; F2= second formant; m: moment

Regarding the OA exercise, we observed no significant difference in the comparison between the moments.

## DISCUSSION

Traditionally, the acoustic analysis used in the evaluation and monitoring of voice disorders is focused on the acoustic inspection (narrow or wideband spectrography) of sustained vowels and/or on the extraction of measurements that allow inferences about the production conditions in the glottal source16. Another perspective of analysis considers that laryngeal alterations co-occur or cause supraglottic adjustments^(10,13)^ and reinforce the importance of extracting acoustic measurements that allow an integrated analysis of the glottic source and the positioning and movement of the vocal tract articulators. Added to this is the fact that the use of the spoken voice is directly related to communication, to the transmission of a message to the interlocutor. Thus, the distortion of the acoustic signal produced by an altered larynx and its repercussions on the articulators can compromise speech intelligibility, either by the loss of acoustic information or by the decrease in distinctiveness in a vowel and/or consonant segments^(24)^.

In this context, one should seek to understand the effect of exercises commonly used in vocal therapy, not only related to changes in the glottic level, but also their potential for changes in supraglottic adjustments and their repercussions on vowel distinctiveness.

When comparing the differences in formant measures before and after five minutes of the different vocal exercises in the MNV, a reduction in the F2 interval of the vowels [a]-[i] was observed only after performing the VSL exercise. Thus, we observed a reduction in the vowel space in the MNV after the VSL. This reduction, noticeable in Figure 2, indicates that these vowel segments are closer to each other, reducing their acoustic distinctiveness, from the reduction in the amplitude of the mouth opening movement and positioning of the mandible in the anteroposterior direction, since the value of F1 is directly related to the posture of the mandible in the vowel quality of a segment, and F2 the main acoustic measure influenced by the shape of the body of the tongue^(3)^.

Possibly, these changes may have been generated by the vocal and laryngeal impact^(25)^ caused by the exercise, as a result of the expected primary effect of this exercise in the glottic region. Added to this is the fact that the presence of a laryngeal lesion can interfere with vowel distinctiveness^(10)^ and that the proposed vocal exercise has a strong influence on tongue mobility, which in turn is related to the F2 values. Consequently, the laryngeal adjustments caused by the vocal exercise effect possibly favored posteriorized tongue posture adjustments and higher tongue dorsum 3.

As for the MVS, it was observed that after five minutes of performing the CAR exercise, we observed a reduction in the interval from F1 for the segments [a]-[i] and [i]-[u], as well as a reduction in the interval from F2 to the segments [a]-[u] and [i]-[u]. This finding may be associated with the reduction of vowel distinctiveness after CAR execution.

Speech through a straw can modify the acoustic impedance of the vocal tract, normally lower than that of the glottis, creating a positive pressure. Thus, the acoustic impedance of the vocal tract can modify the vocal function at two levels: by acoustic-dynamic interaction and by mechanical-acoustic interaction^(21,22)^. This interaction can modify the positioning of the articulators, such as elevation of the soft palate, enlargement of the oropharyngeal region, and greater elevation of the posterior region of the tongue^(26)^. Although the findings of this study, after performing a high-resistance straw in the air, suggest less distinctiveness between vowels, decreasing the amplitude of movements in the anterior region of the oral cavity, they may indicate enlargement of the oropharynx, due to the effect of exercise^(26)^. Since the elevation of the oromandibular complex results in enlargement of the vocal tract close to the glottis, presenting a decrease in formant frequencies^(3)^.

On the other hand, a previous study^(27)^ investigated the CAR and concluded that we observed an improvement in the self-perception of reduced phonatory effort and less vocal fatigue, although there were no changes in other instrumental measures.

Clinically, the exercises investigated in this study can cause changes at the glottic and resonant levels, proven through the selection of acoustic, perceptual and self-assessment measures^(13,19,21,22)^. However, the investigation focused on the vowel space allows us to identify that the strong communication of the glottic source with the resonance boxes can contact the articulators and provoke new articulatory adjustments, with possible influence on the production of the vowel. This fact makes it essential to select exercises associated with the glottic source and vocal tract, together. It is also presumable that the exercise performance time should be investigated by future studies, and in the vocal clinic, therapeutic tests should be carried out through multidimensional voice monitoring.

When performing the comparative analysis between the groups, it was observed that the differences between the vowel spaces, mainly in the F1 interval of the vowels [a]-[i] remained after five minutes of performing the exercise with CAR, with higher values ​​in the MVS. It is also possible to observe that the MVS participants who performed the CAR exercise presented higher values ​​in the F1 interval of the vowels [i]-[u] even before the execution of this exercise. An increase in the difference in vowel space was observed between women from the MNV and MVS after five minutes of the VSL exercise, with a greater interval of F1 and F2 between the vowels [i]-[u].

Thus, our study shows a greater interval in the vowel space of women from the MVS even before the exercise, and that its execution seems to potentiate this difference, especially for the CAR and VSL exercises. . Vocal space reduction was also observed in previous studies, including individuals with unilateral vocal fold paralysis^(9)^, with dysarthria of different etiologies^(4)^ and with dysarthria resulting from Parkinson's disease^(5)^.

Possibly, women with vocal fold nodules have less acoustic distinctiveness compared to vocally healthy women, who seem to benefit differently from the effect of vocal exercises on the positioning of the articulators. We can also infer the greater impact (or perhaps a more immediate effect) of vocal exercises to improve articulatory clarity in individuals without laryngeal lesion compared to individuals with lesion, since an increase in the F1 and F2 intervals between vowels highs and lows directly influences greater speech clarity^(28)^.

In general, we observed the presence of the vowel [i] in all intervals of vowel spaces brought some significant results in the comparison between the MNV and MVS groups. Thus, we can infer that in the production of the vowel [i] the larynx is configured in its highest frontal position^(3)^, with greater tension and stabilization of the larynx, with a lower amplitude of vibration in the vocal folds and high rates of glottal pulses^(29)^. Therefore, the presence of vocal nodules and laryngeal hyperfunction can generate interference in the production of the interval of this vowel in relation to the others.

Regarding the OA exercise, there were no significant changes in any of the groups. In another study, it was observed that the OA exercise produced an immediate positive effect on the vocal aspects and a greater facial expressiveness in patients with Parkinson's disease, especially after 15 minutes of performance^(20)^. In addition, it is found that this exercise, associated with the technique of voiced vibration of the tongue and nasal sound produced an improvement in acoustic measurements and the videostroboscopic examination of the larynx in women without vocal complaints^(30)^. Thus, one can think of two possibilities for these findings: the exercise performance time, which did not cause changes in the vowel space, or the non-association with exercises involving the glottic source, which would contribute to the source-filter coupling.

Therefore, this study presented exploratory evidence from three exercises commonly used in vocal clinic, to expand the critical thinking of clinical speech-language pathologists and researchers about the relationship between laryngeal alteration and articulator positioning.

## LIMITATIONS AND FUTURE DIRECTIONS

Some limiting factors of the study refer to the size of the sample present in each subgroup for the performance of vocal exercises, the rest time between the series of the exercise performed, and the lack of other evaluative parameters in relation to vocal production and perception.

For further studies, it would be interesting to perform the vocal self-perception of the individuals present in the sample and an intelligibility test as a measure of performance performed by listeners, to discard or add information about effort and muscle fatigue. In addition to increasing the sample size and establishing rest time between sets of exercise performed.

## CONCLUSION

The VSL exercise decreases the vowel space in MNV women, with a reduction in F2 values in the vowel interval [a]-[i]. The CAR reduces the vowel space of women in the MVS, with a decrease in F1 values in the intervals [a]-[i] and [i]-[u], and a decrease in F2 values in the intervals [a]-[u] and [i]-[u]. However, MNV women have less vowel space compared to MVS women, before and after performing the CAR exercise. We observed a reduction in the vowel space of the MNV women in relation to the MVS after the VSL exercise. In the analysis of vowel space intervals, the vowel /i/ was the one that most interfered with the results. The OA exercise did not immediately impact the vowel space.

## References

[1] Ladefoged P (2007). Vowels and consonants: an introduction to the sounds of languages..

[2] Pisanski K, Cartei V, Mcgettigan C, Raine J, Reby D (2016). Voice modulation: a window into the origins of human vocal control?. Trends Cogn Sci..

[3] Barbosa PA, Madureira S (2015). Manual de fonética acústica experimental: aplicações a dados do português.

[4] Park EJ, Yoo SD, Kim HS, Lee JH, Yun DH, Kim DH (2019). Correlations between swallowing function and acoustic vowel space in stroke patients with dysarthria. NeuroRehabilitation.

[5] Oliveira M, Pacheco V (2016). Características fonéticas e contrastes fonológicos em dados de fala de pessoas com down: perspectiva da geometria de traços. Lingüística.

[6] Skodda S, Visser W, Schlegel L (2011). Vowel articulation in Parkinson’s disease. J Voice.

[7] Roy N, Leeper HA, Blomgren M, Cameron RM (2001). A description of phonetic, acoustic, and physiological changes associated with improved intelligibility in a speaker with spastic dysarthria. Am J Speech Lang Pathol.

[8] Ishikawa K, Nudelman C, Park S, Ketring C. (2021). Perception and acoustic studies of vowel intelligibility in dysphonic speech. J Voice.

[9] Fauth C, Vaxelaire B, Rodier JF, Volkmar JF, Bouarourou F, Hirsch F (2011). A spatiotemporal prospective study of speech in patients with or without recurrent laryngeal nerve paralysis after thyroid surgery..

[10] França FP, Almeida AA, Lopes LW (2019). Acoustic-articulatory configuration of women with vocal nodules and with healthy voice. CoDAS.

[11] Roy N, Nissen SL, Dromey C, Sapir S (2009). Articulatory changes in muscle tension dysphonia: evidence of vowel space expansion following manual circumlaryngeal therapy. J Commun Disord.

[12] França FP, Evangelista DS, Lopes LW (2017). Revisão sistemática sobre os formantes e a produção da voz e fala. Revista Prolíngua..

[13] Yamasaki R, Murano EZ, Gebrim E, Hachiya A, Montagnoli A, Behlau M (2017). Vocal tract adjustments of dysphonic and non-dysphonic women pre- and post- flexible resonance tube in water exercise: a quantitative mri study. J Voice.

[14] Maxfield L, Palaparthi A, Titze I (2017). New evidence that nonlinear source-filter coupling affects harmonic intensity and f0 stability during instances of harmonics crossing formants. J Voice.

[15] Stemple J, Graze L, Klaben B (2020). Clinical voice pathology: theory and management..

[16] Floro Silva RL, da Silva Antonetti AE, Ribeiro VV, Ramos AC, Brasolotto AG, Silverio KCA (2022). Voiced High-frequency oscillation or lax vox technique? Immediate effects in dysphonic individuals. J Voice.

[17] Oliveira KGSC, Lira ZS, Silva HJ, Lucena JA, Gomes AOC (2020). Oropharyngeal geometry and the singing voice: immediate effect of two semi-occluded vocal tract exercises. J Voice.

[18] Shadle CH, Nam H, Whalen DH (2016). Comparing measurement errors for formants in synthetic and natural vowels. J Acoust Soc Am.

[19] Pimenta RA, Dájer ME, Hachiya A, Tsuji DH, Montagnoli NA (2013). Parâmetros acústicos e quimografia de alta velocidade identificam efeitos imediatos dos exercícios de vibração sonorizada e som basal. CoDAS.

[20] Bento FAM, Diaféria GLA, Fonoff ET, Padovani MMP, Behlau M (2019). Efeito da técnica de sobrearticulação na voz e na fala em indivíduos com doença de Parkinson após cirurgia de estimulação cerebral profunda. Audiol Commun Res.

[21] Paes SM, Behlau M (2017). Dosage dependent effect of high-resistance straw exercise in dysphonic and non-dysphonic women. CoDAS.

[22] Titze IR (2006). Voice training and therapy with a semi-occluded vocal tract: rationale and scientific underpinnings. J Speech Lang Hear Res.

[23] Van Stan JH, Roy N, Awan S, Stemple J, Hillman RE (2015). A taxonomy of voice therapy. Am J Speech Lang Pathol.

[24] Evitts PM, Starmer H, Teets K, Montgomery C, Calhoun L, Schulze A (2016). The impact of dysphonic voices on healthy listeners: listener reaction times, speech intelligibility, and listener comprehension. Am J Speech Lang Pathol.

[25] Azevedo LL, Passaglio KT, Rosseti MB, Silva CB, Oliveira BFV, Costa RC (2010). Avaliação da performance vocal antes e após a vibração sonorizada de língua. Rev Soc Bras Fonoaudiol..

[26] Guzman M, Miranda G, Olavarria C, Madrid S, Muñoz D, Leiva M (2017). Computerized tomography measures during and after artificial lengthening of the vocal tract in subjects with voice disorders. J Voice.

[27] Guzman M, Denizoglu I, Fridman D, Loncon C, Rivas C, García R (2021). Physiologic voice rehabilitation based on water resistance therapy with connected speech in subjects with vocal fatigue. J Voice.

[28] Ferguson SH, Kewley-port D (2007). Talker differences in clear and conversational speech: acoustic characteristics of vowels. J Speech Lang Hear Res.

[29] Kiliç MA, Oğüt F, Dursun G, Okur E, Yildirim I, Midilli R (2004). The effects of vowels on voice perturbation measures. J Voice.

[30] Pereira EC, Silvério KCA, Marques JM, Camargo PAM (2011). Efeito imediato de técnicas vocais em mulheres sem queixa vocal. Rev CEFAC.

