# Supplementary material for: Immediate effect of different exercises in the vocal space of women with and without vocal nodules
Source: Codas. 2022 Jul 22;34(5):e20210157. doi: 10.1590/2317-1782/20212021157en (PMC9886185; doi:10.1590/2317-1782/20212021157en)
Supplement: Translation

# Translation: Efeito imediato de diferentes exercícios no espaço vocálico de mulheres com e sem nódulos vocais

**DOI:** 10.1590/2317-1782/20212021157pt

**Authors:** Fernanda Pereira França, Anna Alice Almeida, Leonardo Wanderley Lopes

**Affiliations:** 1 Universidade Federal da Paraíba – UFPB, João Pessoa, PB, Brasil.

**Keywords:** Voz, Distúrbios da Voz, Acústica, Doenças da laringe, Treinamento da Voz

## Abstract

**Objetivo:**

Investigar o efeito imediato da vibração sonorizada de língua (VSL), do canudo de alta resistência no ar (CAR) e da sobrearticulação (SA) sobre o espaço vocálico de mulheres vocalmente saudáveis (MVS) e com nódulos vocais (MNV).

**Método:**

Participaram 12 mulheres no MNV e 12 mulheres no MVS, alocadas para execução dos exercícios vocais de VSL, CAR e AS. Cada participante realizou apenas um dos três exercícios propostos, durante 5 minutos, antecedidos e sucedidos pela gravação de uma sequência de frases-veículo para extração dos formantes (F1 e F2) dos segmentos vocálicos [a, i, u]. O espaço vocálico foi analisado por meio das diferenças entre as medidas dos formantes das vogais.

**Resultados:**

houve redução de F1 no intervalo [a]-[i] e [i]-[u] e de F2 entre as vogais [a]-[u] e [i]-[u] no MVS, após realização do CAR. No MNV, observou-se redução de F2 no intervalo [a]-[i] após VSL. Na análise intergrupo, houve maiores valores de F1 entre os intervalos das vogais [a]-[i] e [i]-[u] no MVS, antes da realização do CAR, e após exercício apenas no intervalo [a]-[i]. Observou-se maior valor de F1 e F2 no intervalo entre as vogais [i]-[u] no MNV após VSL.

**Conclusão:**

O exercício de VSL diminuiu o espaço vocálico em mulheres do MNV. O CAR reduziu o espaço vocálico de mulheres do MVS. O MNV apresentou menor espaço vocálico em relação ao MVS, antes e após a realização do CAR. Houve redução do espaço vocálico no MNV em relação ao MNV após o exercício de VSL.

## INTRODUÇÃO

As medidas formânticas são os principais correlatos acústicos associados à descrição de segmentos vocálicos. Os formantes são o resultado dos pulsos de ar que passam pelas pregas vocais, fazem vibrar as paredes do trato vocal produzindo zonas de concentração de energia^(1)^.

A partir da análise dos formantes, pode-se investigar a movimentação e o posicionamento dos articuladores (mandíbula, lábios e língua) durante a fala. Nesse sentido, diferentes combinações no posicionamento desses articuladores contribuem para a distintividade acústico-articulatória dos segmentos vocálicos^(2)^.

As vogais [a, i, u] são as que mais se distinguem acusticamente, uma vez que se encontram nas extremidades do triângulo articulatório^(1)^. De modo geral, a língua encontra-se em posição mais elevada na produção das vogais [i] e [u], o que contribui para os menores valores da frequência do primeiro formante (F1) para essas vogais. Por sua vez, o complexo oromandibular tende a encontrar-se mais abaixado na produção da vogal [a], o que pode ocasionar a elevação da frequência de F1^(1)^. O segundo formante (F2) tem valores de frequência mais elevados na vogal [i], justificado pela posição mais anterior da língua. A frequência de F2 tende a ser mais baixa na vogal [u], devido ao deslocamento posterior da língua, e assume valores intermediários entre as vogais [i] e [u] na produção da vogal [a]^(1,3)^.

O triângulo formado pelas frequências dos formantes das vogais pode ser, então, representado por um método gráfico através de um diagrama que une duas coordenadas, de acordo com valor do primeiro e segundo formante, sendo a F2 na abscissa e a F1 na ordenada. A área criada internamente ao triângulo é denominada espaço vocálico, a partir da distância Euclidiana entre as vogais [i] – [a] – [u]. Um espaço ampliado desse triângulo pode ser um dos marcadores de maior distintividade acústica entre as vogais e, consequentemente, maior amplitude de movimentação dos articuladores, enquanto a redução do espaço vocálico pode indicar o oposto. Acrescenta-se a isso o fato de que a distintividade vocálica é um dos fatores responsáveis pela inteligibilidade de fala, especificamente no que diz respeito às vogais de uma dada língua^(3)^.

Tradicionalmente, as medidas formânticas têm sido utilizadas para diferentes finalidades, o que inclui a caracterização do espaço vocálico em diferentes populações em função do gênero, idade, variação linguística, estilos de fala e indivíduos com distúrbios da comunicação de diferentes etiologias. Por exemplo, o espaço vocálico tem sido utilizado para avaliar a relação entre a articulação das vogais e a inteligibilidade de fala em indivíduos com doença de Parkinson, com sequela de acidente vascular encefálico, em crianças com síndrome de Down, com fissura palatina, entre outros^(4-6)^. Nesse sentido, observa-se que em pacientes disártricos, há redução do espaço vocálico devido à diminuição na amplitude e velocidade dos movimentos dos articuladores. Um dos principais efeitos do tratamento nesses pacientes é a expansão do espaço vocálico^(7)^.

A presença de um distúrbio de voz também pode interferir na inteligibilidade de fala e na área do espaço vocálico. Indivíduos disfônicos podem apresentar menor inteligibilidade de fala^(8)^, redução do espaço vocálico^(9)^, com menores valores de F1 e F2 para as vogais orais [a], [i] e [u] do português brasileiro em relação a mulheres vocalmente saudáveis^(10)^. Além disso, observou-se ampliação do espaço vocálico em pacientes com disfonia por tensão muscular primária após tratamento com terapia manual circunlaríngea^(11)^.

Dessa forma, sabe-se que a modificação da atividade laríngea pela presença de uma alteração funcional ou estrutural pode repercutir em ajustes supraglóticos disfuncionais (tais como constrição supraglótica, redução do espaço orofaríngeo e elevação-anteriorização do complexo hiolaríngeo) que coocorrem ou são consequência de tais alterações^(10,12)^. Nesse contexto, os exercícios vocais são utilizados no processo de reabilitação vocal para possível modificação dos ajustes disfuncionais, ocasionando efeitos com diferentes focos, seja na fonte glótica, no trato vocal ou na interação entre a fonte glótica e o trato vocal^(13,14)^.

A seleção dos exercícios na clínica vocal deve então ser direcionada de acordo com a queixa principal ou necessidades do paciente, seu comportamento vocal e sua demanda vocal. Os objetivos principais dos exercícios vocais incluem: reequilibrar os subsistemas envolvidos na produção vocal; melhorar o equilíbrio, o tônus e a resistência dos músculos envolvidos na produção vocal; promover o desenvolvimento de um movimento mucoondulatório saudável das pregas vocais e reforçar novos comportamentos relacionados ao uso da voz^(15)^.

No entanto, a análise acústica, com utilização de medidas de perturbação, energia e ruído relacionadas mais estritamente à fonte glótica, é o principal padrão de referência utilizada para monitorar o efeito imediato da intervenção clínica^(16)^. Embora as medidas formânticas tenham sido amplamente investigadas relacionadas à produção vocal de indivíduos vocalmente saudáveis ou com distúrbio de voz, o principal interesse esteve relacionado ao processo de identificação, caracterização do distúrbio ou confirmação/diferenciação diagnóstica da alteração vocal e laríngea.

Nos últimos anos, a ressonância funcional magnética^(13)^ e a faringometria^(17)^ acústica têm incluído a investigação do efeito dos exercícios vocais na região supraglótica. Deve-se considerar que essas tecnologias citadas não são acessíveis para uso na rotina clínica e que o estudo das medidas formânticas, especificamente, do espaço vocálico pré e pós-intervenção^(11)^, podem fornecer inferências acerca do efeito dos exercícios vocais sobre o posicionamento e a movimentação dos articuladores pré e pós exercício vocal. Além disso, as medidas formânticas são consideradas o principal padrão de referência para validar os estudos de imagem relacionados ao trato vocal e para construção de modelos físicos para síntese de vozes humanas^(18)^.

Sendo assim, a pergunta de pesquisa que motivou a realização deste estudo pode ser definida da seguinte forma: será que o foco de ação primário dos exercícios vocais (fonte glótica, acoplamento fonte-filtro ou amplitude de movimentação dos articuladores) ocasiona diferentes efeitos sobre o espaço vocálico das vogais orais [a, i, u] em mulheres vocalmente saudáveis e com nódulos vocais?

Nesse contexto, o objetivo desde estudo foi investigar o efeito imediato da vibração sonorizada de língua (VSL), canudo de alta resistência no ar (CAR) e sobrearticulação (AS) sobre o espaço vocálico de mulheres vocalmente saudáveis e com nódulos vocais.

## MÉTODO

### Desenho do estudo

Trata-se de um estudo analítico e de intervenção. Foi avaliado e aprovado pelo Comitê de Ética em Pesquisa da Instituição de origem, com o parecer número 2.158.960. Todos os participantes receberam explicação sobre a pesquisa e assinaram o termo de consentimento livre e esclarecido.

### Amostra

Para realização desta pesquisa foram constituídos dois grupos: mulheres com nódulos vocais (grupo MNV), com a participação de mulheres adultas atendidas no Laboratório de Voz de uma Instituição de Ensino Superior; e o grupo mulheres vocalmente saldáveis (MVS), formado por mulheres adultas sem distúrbio de voz. Para o MNV foram considerados os seguintes critérios de elegibilidade:

- Apresentar laudo otorrinolaringológico de nódulos vocais;
- Ser do sexo feminino, devido a maior prevalência de distúrbios da voz nesse gênero e pela influência dessa variável nos valores da média da frequência fundamental (f0) e das medidas formânticas, que apresentam diferenças entre homens e mulheres adultos, devido às características anatômicas das pregas vocais e do trato vocal;
- Idade 19 e 59 anos, para evitar as modificações na fonte glótica e no trato vocal relativas à infância, adolescência e senescência;

Para a composição do grupo mulheres vocalmente saldáveis (MVS), foram recrutadas ativamente mulheres funcionárias e alunas de um curso de graduação da Instituição de Ensino Superior onde foi realizada a pesquisa, que se disponibilizassem para a realização da mesma e, que se enquadrassem nos mesmos critérios de elegibilidade citados para o MNV, com exceção do diagnóstico de nódulos vocais e:

- Não apresentar queixa vocal no momento da coleta ou nos seis meses anteriores, respondendo negativamente à pergunta “você apresenta um problema de voz atualmente ou nos últimos seis meses?”
- Apresentar laudo otorrinolaringológico de laringe normal;

Em ambos os grupos foram considerados os seguintes critérios de exclusão:

- Apresentar infecções de vias áreas superiores no momento da gravação, o que geraria modificação nas cavidades de ressonância e, consequentemente, nas medidas formânticas;
- Possuir frênulo lingual encurtado, disfunção temporomandibular, e/ou alterações estruturais e funcionais dos articuladores, o que modificaria os ajustes do trato vocal supraglótico;
- Ter alterações cognitivas ou neurológicas que impedissem a realização dos procedimentos de coleta;
- Ter realizado terapia fonoaudiológica anteriormente.

A variável faixa etária foi utilizada para o pareamento do MVS com o MNV, considerando uma variação da idade de cinco anos para mais ou para menos, seguindo uma proporção de um controle para cada caso (1:1).

Todas as mulheres do MNV foram recrutadas no momento da consulta inicial no Laboratório onde foi realizada a pesquisa, antes de iniciar o processo de terapia vocal propriamente dita. Inicialmente, os pesquisadores tiveram acesso à ficha de avaliação das pacientes para verificação dos dados sociodemográficos (idade) e do diagnóstico laríngeo feito pelo médico otorrinolaringologista previamente à consulta inicial para avaliação vocal, conforme rotina do referido Laboratório. Aquelas que cumpriam os critérios de elegibilidade definidos foram abordadas ao final da sessão de avaliação vocal e convidadas a participar da pesquisa, após leitura e concordância com o TCLE. Dessa forma, a amostra do MNV foi composta por 12 mulheres com média de idade de 36, 47 ± 12,22 anos.

Para recrutamento das mulheres do MVS, considerou-se, então, a observação da faixa etária das participantes do MNV. Sendo assim, elas foram contatadas pelo pesquisador, que fornecia informações sobre a pesquisa e, em caso de anuência da voluntária e assinatura do TCLE, procedia-se com a coleta de informações em relação à idade e presença de queixa vocal. Aquelas que correspondiam aos critérios de elegibilidade para o MVS eram encaminhadas para realização do exame visual laríngeo (videolaringoscopia) com médico otorrinolaringologista para descartar a presença de alteração funcional ou estrutural na laringe. Após os resultados dos exames, as mulheres com laudo de “laringe normal” foram recrutadas para dar continuidade na participação do estudo. Dessa forma, a amostra do MVS foi composta por 12 mulheres com média de idade de 33, 86 ± 11,59 anos.

### Procedimentos da coleta de dados

As participantes do MNV e MVS selecionadas a partir dos critérios de elegibilidade foram convidadas a participar de uma sessão para coleta de voz e realização da intervenção. Ao iniciar a sessão, foram retomados os objetivos da pesquisa a partir da leitura do TCLE e confirmados os dados pessoais, incluindo.

A coleta de dados ocorreu em três etapas, a saber: 1) avaliação das estruturas do sistema estomatognático e coleta inicial das tarefas de fala; 2) realização dos exercícios vocais; 3) e coleta de voz pós exercício vocal.

**Etapa 1** - Avaliação das estruturas do sistema estomatognático e coleta inicial das tarefas de fala.

Inicialmente, foi realizada avaliação das estruturas do sistema estomatognático, observando-se: morfologia e mobilidade de lábios, língua, bochechas e palato mole; tonicidade de lábios, língua e bochechas; amplitude de abertura da boca e presença de queixas relacionadas à articulação temporomandibular; e a presença queixa de infecções de vias aéreas superiores (de acordo com o autorrelato). O objetivo dessa avaliação foi descartar a presença de desordem temporomandibular, alteração no frênulo lingual, ou qualquer alteração estrutural e funcional que poderia influenciar nos resultados desse estudo, em função da interferência dessas alterações sobre os ajustes articulatórios.

Posteriormente, procedeu-se com a gravação das tarefas de fala. Para tanto, utilizou-se o *software* Fonoview, versão 4.5, da CTS Informática, *desktop* Dell *all-in-one*, microfone cardioide unidirecional, da marca Senheiser, modelo E- 835, localizado em um pedestal e acoplado a um pré-amplificador Behringer, modelo U-Phoria UMC 204. As vozes foram coletadas em cabine de gravação, em um laboratório de estudos da voz, com tratamento acústico e ruído inferior a 50 dB NPS, verificado com medidor de nível de pressão sonora R8050 da *Reed Instruments*; com taxa de amostragem de 44000 Hz,16 bits por amostra e distância de 10 cm entre o microfone e a boca do falante.

Para a coleta das vozes, as mulheres ficaram em pé, situando o pedestal a sua frente, de acordo com a distância preconizada entre a boca e o microfone, conforme descrito acima. Elas foram instruídas a realizar uma inspiração de modo habitual e, na sequência, repetir por três vezes cada uma das seguintes frases-veículo, separadamente: “Digo p **a** pa baixinho”, “Digo p **i** pa baixinho” e “Digo p **u** pa baixinho”.

As frases citadas contêm os segmentos vocálicos [a, i, u] em contexto CV (consoante - vogal), em sílaba inicial de palavra, não acentuada, com vogal antecedida e sucedida do fonema oclusivo bilabial desvozeado. A escolha dessa frase justifica-se pela pouca influência que estas consoantes têm sobre os formantes das vogais adjacentes^(3)^ e pela necessidade de se homogeneizar o contexto fonético para obtenção de todas as vogais das amostras de fala. Dessa forma, houve o mínimo controle dos aspectos prosódicos, sem interferências na realização dos sons vocálicos na investigação da distintividade acústica das vogais.

As vogais [a, i, u] foram escolhidas devido à reconhecida distintividade acústica entre elas, formando um triângulo articulatório vocálico em suas extremidades^(3)^. Além disso, elas obedecem a um padrão formântico de consenso entre os pesquisadores, que corresponde às características típicas de vogais que apresentam o máximo e o mínimo de abertura vocálica e de movimento de recuo e de avanço, de abaixamento e levantamento da língua.

**Etapa 2** - Realização dos exercícios vocais

Após essa coleta inicial das tarefas de fala, as informantes foram orientadas quanto aos próximos procedimentos experimentais da pesquisa, relativos à execução dos exercícios vocais. Para esta pesquisa foram escolhidos três exercícios frequentemente utilizados em terapia vocal e amplamente citados na literatura da área^(14,19,20)^, incluindo a VSL, o CAR e a SA.

Para seleção desses exercícios foram utilizados os seguintes critérios:

- Exercícios utilizados rotineiramente em terapia vocal e citados em estudos de intervenção fonoaudiológica na reabilitação dos distúrbios da voz^(21,22)^;
- Exercícios cujos princípios fisiológicos de ação descritos na literatura da área envolvessem efeitos definidos e, na medida do possível, independentes, quanto à fonte glótica^(19)^, melhora da interação fonte-filtro^(14)^ e modificação na movimentação e posicionamento dos articuladores^(20)^.

De acordo com a taxonomia das abordagens utilizadas em terapia vocal^(23)^, a VSL é considerada um exercício classificado dentro da categoria função vocal, caracterizado como um tipo de intervenção que direciona a atenção do paciente para modificação de ajustes fonatórios (glóticos) propriamente ditos; o exercício de CAR classificado na categoria de abordagem somatossensorial, cujo objetivo principal é direcionar a atenção do paciente para a modificação da entrada somática; e a SA é classificada dentro da abordagem musculoesquelética, direcionando a atenção do paciente para a modificação muscular relacionada aos movimentos das estruturas da cavidade oral.

Dessa forma, os três exercícios enfocam basicamente a fonte glótica (VSL), a movimentação dos articuladores do trato vocal (SA) e o acoplamento da fonte e do trato vocal (CAR). O estudo dessas técnicas favorecerá a compreensão da interação entre a fonte sonora e os mecanismos de articulação do som, descrevendo as influências destas para a comunicação, assim como reproduzindo as propriedades físicas inerentes à produção de fala^(10,12)^.

Inicialmente, foram apresentadas as três técnicas às participantes e solicitado que executassem uma vez cada uma delas. Conforme percebíamos a facilidade de execução ou a partir do relato do indivíduo quanto à dificuldade de execução, escolhíamos a técnica que seria executada por essa voluntária, preconizando a técnica produzida com maior facilidade e menor esforço pela participante. O objetivo desse procedimento foi excluir a possibilidade de que a dificuldade na execução influenciasse no efeito da técnica. Caso a participante apresentasse esses requisitos em mais de um exercício, a alocação seria realizada por conveniência, para compor a paridade dos grupos. Desse modo, as participantes do MNV e do MVS foram alocadas em três grupos (A, B e C), a depender do exercício vocal executado. Cada voluntária participou, necessariamente, de apenas um dos grupos.

O grupo A, composto por quatro mulheres do MNV (média de idade ±35 anos) e quatro mulheres do MVS (média de idade ±30,75 anos), executou o exercício de VSL. Nesse sentido, as participantes foram solicitadas a emitir o som do fonema [ɾ] de forma sustentada em frequência e intensidade confortável autorreferida. A escolha deste exercício deve-se ao fato de que ele pode viabilizar modificações na fonte glótica, com influência direta sobre a fisiologia das pregas vocais^(19)^. A realização dessa técnica auxilia na reabsorção das lesões benignas de pregas vocais, modificando o quadro hipercinético encontrado nos casos de nódulos vocais, resultando no aumento da irrigação sanguínea nas pregas vocais, diminuindo as resistências elásticas e viscosas.

O grupo B, composto por cinco mulheres do MNV (média de idade ±36 anos) e cinco mulheres do MVS (média de idade ±35,8 anos), realizou o exercício com CAR. Para a realização desse exercício foi utilizado um canudo de plástico rígido, com comprimento de 8,7 cm e diâmetro de 1,5 mm. As integrantes dos grupos foram orientadas a emitir um som semelhante ao [vu] de modo sustentado em frequência e intensidade confortável autorreferida. Elas foram orientadas que todo o fluxo sonoro saísse pelo canudo, conforme demonstrado pelo pesquisador.

A escolha desse exercício foi justificada pelo efeito que ele ocasiona no sistema de produção vocal, realizando ajustes nas pregas vocais e no trato vocal, aumentando a interação entre a fonte glótica e o trato vocal supraglótico^(21)^. As pregas vocais vibram de forma mais suave, pois são levemente separadas (abduzidas) pela ação da pressão que fecha a saída de ar gerada dentro do “tubo”. O fluxo aéreo também é reduzido, bem como o impacto do contato entre as pregas vocais, auxiliando também na reabsorção da lesão de massa entre as pregas vocais^(22)^.

O grupo C, composto por três mulheres do MNV (média de idade ±34 anos) e três mulheres do MVS (média de idade ±34,66), executaram o exercício de SA. Quanto ao exercício de SA, as integrantes do grupo foram orientadas para realizar exagero nos movimentos articulatórios, fazendo uma maior excursão muscular, com grande abertura de boca e maior amplitude de movimentação labial, durante a leitura de um texto foneticamente balanceado, baseado na Brazilian Portuguese version of Vocal Profile Analysis Scheme – PB-VPAS (2007).

A escolha do exercício de SA foi baseada na fisiologia encontrada durante sua realização, uma vez que envolve a movimentação dos articuladores, focando sua atuação no trato vocal, porém com a finalidade de diminuir a hipertonicidade laríngea, melhorando a articulação da fala e a projeção vocal.

Na sequência, para cada exercício vocal foi realizada 5 séries de 1 minuto, totalizando 5 minutos de execução^(21)^, com gravação vocal das três frases-veículo descritas anteriormente, antes e após os cinco minutos de execução.

As participantes do grupo MVS foram recrutadas após a coleta com o grupo MNV. Tal procedimento foi definido para favorecer a mesma quantidade de informantes em ambos os grupos.

A extração do primeiro e segundo formante das vogais [a, i, u] foi realizada no *software* Praat, versão 5.3.77h, a partir da representação da vogal em um espectrograma de banda larga. O Praat é uma ferramenta para a análise de voz, desenvolvida por Paul Boersma e David Weenink, do *Institute of Phonetic Sciences*, Universidade de Amesterdã.

A partir da seleção e da segmentação dos sons vocálicos em contextos CV, foi possível obter a média das medidas acústicas investigadas. Para a extração da média dos formantes no Praat, selecionou-se a opção denominada de *Formant*, obtendo o valor numérico de F1, F2 e F3 expresso em Hertz (Hz).

### Procedimento de análise de dados

Para a análise e discussão dos dados, a execução do exercício foi considerada da seguinte forma: m0 – antes da realização do exercício vocal; m5 – após o quinto minuto de execução do exercício vocal.

A análise estatística foi realizada considerando as medidas descritivas, como média e desvio-padrão das diferenças entre as vogais (espaço das vogais), ou seja, o resultado da subtração entre as médias das vogais [a]-[u]; [a]-[i] e [i]-[u], com relação ao primeiro e ao segundo formante.

Para as comparações intragrupo (MNV e MVS) entre os intervalos dos espaços das vogais [a]-[u], [a]-[i] e [i]-[u] de F1 e F2, foram utilizados os testes t de *Student* pareado e *Wilcoxon* para dados pareados. Quando não satisfeita à suposição de normalidade, o teste t foi substituído pelo não paramétrico de *Wilcoxon*. Na comparação intergrupo (MVS vs. MNV) foram utilizados os testes t de *Student* e o teste não paramétrico de *Wilcoxon*, considerando amostras independentes.

Todas as análises foram realizadas no *software* R. O nível de significância considerado foi de 5%.

## RESULTADOS

Na Tabela 1 podem ser observadas as médias e o desvio-padrão das diferenças de F1 e F2 das vogais, entre os intervalos [a]-[u], [a]-[i] e [i]-[u] nos momentos m0 e m5 de todas as técnicas vocais investigadas. Esta também expõe dados da análise comparativa intragrupo, com base nas médias das diferenças vocálicas de F1 e F2, de acordo com cada intervalo vocálico analisado, antes e após a realização dos exercícios vocais.

**Tabela 1:** Média e desvio-padrão das diferenças dos intervalos vocálicos e comparação **intragrupo** entre as diferenças de F1 e F2 das vogais nos momentos pré e pós 5 minutos

**Exercício**		**MNV**								
		**[a] – [u]**			**[a] – [i]**			**[i] – [u]**		
		**Tempo m0**	**Tempo m5**	**p-valor**	**Tempo m0**	**Tempo m5**	**p-valor**	**Tempo m0**	**Tempo m5**	**p-valor**
		**Média DP**	**Média DP**		**Média DP**	**Média DP**		**Média DP**	**Média DP**	
Vibração de língua	F1	533,08±182,57	397,74±107,21	0,1869	500,45±150,72	416,15±120,90	0,1083	32,63±103,93	18,40±17,98	0,1083
	F2	682,44±204,27	654,2±121,80	0,775	1153,32±132,90	1021,81±190,52	0,0274	1835,76±322,39	1676,01±253,65	0,0857
Canudo alta resistência	F1	350,05±154,38	344±109,92	0,8533	375,24±150,75	372,11±98,29	0,9303	25,19±8,41	28,11±29,56	0,8386
	F2	562,93±296,95	647,10±198,89	0,5448	1078,63±279,35	1027,80±287,64	0,4509	1641,56±425,82	1674,90±291,13	0,693
Sobrearticulação	F1	447,34±86,17	458,8±39,26	0,8222	512,62±114,15	560,35±62,24	0,2912	65,28±42,87	101,55±79,67	0,2348
	F2	761,10±89,08	634,66±203,23	0,531	1120,59±64,25	1206,87±130,93	0,1675	1881,69±45,97	1841,50±333,48	0,8652
**Exercício**		MVS								
		**[a] – [u]**			**[a] – [i]**			**[i] – [u]**		
		**Tempo m0**	**Tempo m5**	**p-valor**	**Tempo m0**	**Tempo m5**	**p-valor**	**Tempo m0**	**Tempo m5**	**p-valor**
		**Média DP**	**Média DP**		**Média DP**	**Média DP**		**Média DP**	**Média DP**	
Vibração de língua	F1	433,07±33,53	385,18±85,89	0,3776	503,39±40,66	475,74±116,76	0,6224	70,32±38,02	90,56±44,27	0,4355
	F2	680,42±94,69	631,73±47,87	0,3709	1002,12±91,60	1042,42±61,66	0,1681	1682,55±165,62	1674,15±75,07	0,9081
Canudo alta resistência	F1	491,57±59,06	443,42±40,80	0,1578	601,85±68,16	513,53±73,98	0,0251	110,27±69,67	70,10±50,33	0,0498
	F2	755,13±78,37	697,72±72,27	0,0463	1195,64±100,49	1173,79±26,60	0,6138	1950,78±100,21	1871,52±57,68	0,0419
Sobrearticulação	F1	480,15±98,72	496,08±84,37	0,7019	541,55±110,48	552,67±87,58	0,8038	61,40±18,13	56,59±13,08	0,302
	F2	815,17±115,34	772,93±70,67	0,2833	1098,24±90,67	1135,99±70	0,2833	1913,41±47,31	1908,92±127,90	0,9424

Valores significativos (p<0,05) Teste t de pareado; Wilcoxon pareado

Legenda: MNV = grupo mulheres com nódulos vocais; MVS = grupo mulheres vocalmente saudáveis; DP = desvio padrão; F1= primeiro formante; F2= segundo formante; m: momento

No grupo MNV, observa-se que há diferença significativa para o F2 após execução do exercício de VSL nos intervalos das vogais [a]-[i] (p = 0,0274) (Tabela 1). Houve redução dos intervalos dessas vogais após realização do exercício. Na Figura 1 pode-se verificar a diminuição do espaço vocálico entre essas vogais na condição especificada para o MNV.

**Figura 1:** Triângulo acústico-articulatório das vogais [a], [i] e [u] do grupo MNV do pré e pós 5 minutos do exercício de VSL

Com relação ao grupo MVS, após realização do exercício CAR, houve diferença significativa para o F1 entre os intervalos das vogais [a]-[i] (p= 0,0251) e [i]-[u] (p = 0,0498), e para o F2 entre os intervalos das vogais [a]-[u] (p = 0,0463) e [i]-[u] (p = 0,0419) (Tabela 1). Houve redução dos intervalos dessas vogais após realização do exercício. Na Figura 2 pode-se verificar a diminuição do espaço vocálico entre essas vogais na condição especificada para o MVS.

**Figura 2:** Triângulo acústico-articulatório das vogais [a], [i] e [u] do grupo MVS do pré e após 5 minutos do exercício com CAR

Na sequência, a Tabela 2 exibe análise de comparação entre o MNV e MVS quanto ao intervalo vocálico com base em F1 e F2, nos momentos m0 e m5 de todas as técnicas vocais investigadas. Antes da realização dos exercícios, observou-se diferença para F1 entre o intervalo [a]-[i] (p = 0,02436) e [i]-[u] (p = 0,04999) no grupo CAR. É possível observar menores valores entre esses intervalos no grupo MNV. Na Figura 3 pode-se verificar a diminuição do espaço vocálico neste grupo. Após a realização do exercício VSL, houve diferença entre os grupos para F1(p = 0,0396) e F2 (p = 0,0285) entre o intervalo [i]-[u]. E após execução do exercício CAR, observou-se diferença entre os grupos para F1, com relação ao intervalo [a]-[i] (p = 0,03517). Observa-se que após a realização do VSL e CAR, mulheres com nódulos vocais, de ambos os grupos, apresentam redução dos valores nesses intervalos mencionados. A Figura 4 e 5 expõe a diminuição do espaço vocálico no MNV na condição especificada para o grupo que realizou o VSL e o CAR, respectivamente.

**Tabela 2:** Média e desvio-padrão das diferenças dos intervalos vocálicos e comparação **intergrupo** entre as diferenças de F1 e F2 das vogais nos momentos pré e pós 5 minutos

**Exercício**		**Tempo m0**								
		**[a] – [u]**			**[a] – [i]**			**[i] – [u]**		
		**MNV**	**MVS**	**p-valor**	**MNV**	**MVS**	**p-valor**	**MNV**	**MVS**	**p-valor**
		**Média DP**	**Média DP**		**Média DP**	**Média DP**		**Média DP**	**Média DP**	
**Vibração de língua**	**F1**	533,08±182,57	433,07±33,53	0,3556	500,45±150,72	503,39±40,66	0,9720	32,63±103,93	70,32±38,02	0,1403
	**F2**	682,44±204,27	680,42±94,69	0,9865	1153,32±132,90	1002,12±91,60	0,1163	1835,76±322,39	1682,55±165,62	0,4372
**Canudo alta resistência**	**F1**	350,05±154,38	491,57±59,06	0,1121	375,24±150,75	601,85±68,16	**0,0243**	25,19±8,41	110,27±69,67	**0,0499**
	**F2**	562,93±296,95	755,13±78,37	0,2260	1078,63±279,35	1195,64±100,49	0,4184	1641,56±425,82	1950,78±100,21	0,1821
**Sobrearticulação**	**F1**	447,34±86,17	480,15±98,72	0,6873	512,62±114,15	541,55±110,48	0,7683	65,28±42,87	61,40±18,13	0,8953
	**F2**	761,10±89,08	815,17±115,34	0,5576	1120,59±64,25	1098,24±90,67	0,7469	1881,69±45,97	1913,41±47,31	0,4519
**Exercício**	**Tempo m5**								
		**[a] – [u]**			**[a] – [i]**			**[i] – [u]**		
		**MNV**	**MVS**	**p-valor**	**MNV**	**MVS**	**p-valor**	**MNV**	**MVS**	**p-valor**
		**Média DP**	**Média DP**		**Média DP**	**Média DP**		**Média DP**	**Média DP**	
**Vibração de língua**	**F1**	397,74±107,21	385,18±85,89	0,8612	416,15±120,90	475,74±116,76	0,5049	18,40±17,98	90,56±44,27	**0,0396**
	**F2**	654,2±121,80	631,73±47,87	0,7490	1021,81±190,52	1042,42±61,66	0,8479	1727,2±253,65	1794,5±75,07	**0,0285**
**Canudo alta resistência**	**F1**	344±109,92	443,42±40,80	0,1155	372,11±98,29	513,53±73,98	**0,0351**	28,11±29,56	70,10±50,33	0,1553
	**F2**	647,10±198,89	697,72±72,27	0,6155	1027,80±287,64	1173,79±26,60	0,5476	1674,90±291,13	1871,52±57,68	0,2076
**Sobrearticulação**	**F1**	458,8±39,26	496,08±84,37	0,5404	560,35±62,24	552,67±87,58	0,9081	101,55±79,67	56,59±13,08	0,7000
	**F2**	634,66±203,23	772,93±70,67	0,4000	1206,87±130,93	1135,99±70	0,3619	1841,50±333,48	1908,92±127,90	0,7684

Valores significativos (p<0,05) Teste t de pareado; Wilcoxon pareado

Legenda: MNV = grupo mulheres com nódulos vocais; MVS = grupo mulheres vocalmente saudáveis; DP = desvio padrão; F1= primeiro formante; F2= segundo formante; m: momento

**Figura 3:** Triângulo acústico-articulatório das vogais [a], [i] e [u] do grupo MNV e MVS antes da realização do exercício com CAR

**Figura 4:** Triângulo acústico-articulatório das vogais [a], [i] e [u] do grupo MNV e MVS após 5 min. da realização do exercício de VSL

**Figura 5:** Triângulo acústico-articulatório das vogais [a], [i] e [u] do grupo MNV e MVS após 5 min. da realização do exercício com CAR

Com relação ao exercício SA, não houve diferença significativa na comparação entre os momentos.

## DISCUSSÃO

Tradicionalmente, a análise acústica utilizada na avaliação e monitoramento dos distúrbios da voz é focada na inspeção acústica (espectrografia de faixa estreita ou larga) de vogais sustentadas e/ou na extração de medidas que possibilitam inferências acerca das condições de produção na fonte glótica^(16)^. Uma outra perspectiva de análise, considera que as alterações laríngeas coocorrem ou ocasionam ajustes supraglóticos^(10,13)^ e reforçam a importância de extração de medidas acústicas que possibilitem uma análise integrada da fonte glótica e do posicionamento e movimentação dos articuladores do trato vocal. Acrescenta-se a isso o fato de que o uso da voz falada está diretamente relacionado à comunicação, à transmissão de uma mensagem ao interlocutor. Dessa forma, a distorção do sinal acústico produzido por uma laringe alterada e suas repercussões nos articuladores podem comprometer a inteligibilidade de fala, seja pela perda da informação acústica ou pela diminuição da distintividade em segmentos vocálicos e/ou consonantais^(24)^.

Nesse contexto, deve-se buscar compreender o efeito dos exercícios comumente utilizados na terapia vocal não somente relacionados às modificações no nível glótico, mas também o seu potencial de modificações de ajustes supraglóticos e suas repercussões na distintividade vocálica.

Ao comparar as diferenças das medidas formânticas pré e pós cinco minutos dos diferentes exercícios vocais no MNV, observou-se redução do intervalo de F2 das vogais [a]-[i] apenas após execução do exercício de VSL. Dessa forma, houve redução do espaço vocálico no MNV após a VSL. Essa redução, perceptível na Figura 2, indica que esses segmentos vocálicos estão mais próximos entre si, diminuindo sua distintividade acústica, a partir da redução da amplitude do movimento de abertura de boca e posicionamento de mandíbula no sentido anteroposterior, uma vez que, o valor de F1 está diretamente relacionado à postura da mandíbula na qualidade vocálica de um segmento, e F2 a principal medida acústica influenciada pela forma do corpo da língua^(3)^.

Possivelmente, essas modificações podem ter sido geradas pelo impacto vocal e laríngeo^(25)^ provocados pelo exercício, como resultado do efeito primário esperado desse exercício na região glótica. Acrescenta-se a isso o fato de que a presença de lesão laríngea pode interferir na distintividade vocálica^(10)^ e que o exercício vocal proposto tem forte influência na mobilidade de língua, o que por sua vez tem relação com os valores de F2. Consequentemente, os ajustes laríngeos ocasionados pelo efeito do exercício vocal, possivelmente, favoreceram ajustes de postura de língua posteriorizada e dorso de língua mais elevado^(3)^.

Quanto ao MVS, observou-se que após cinco minutos de execução do exercício de CAR houve redução do intervalo de F1 para os segmentos [a]-[i] e [i]-[u], assim como redução do intervalo de F2 para os segmentos [a]-[u] e [i]-[u]. Tal achado pode ser associado à redução da distintividade vocálica após execução do CAR.

A fonação em canudo pode modificar a impedância acústica do trato vocal, normalmente mais baixa que a da glote, criando uma pressão positiva. Dessa forma, a impedância acústica do trato vocal pode modificar a função vocal em dois níveis: por interação acústico-dinâmica e por interação mecânico-acústica^(21,22)^. Essa interação pode modificar o posicionamento dos articuladores, como a elevação do véu palatino, ampliação da região orofaríngea, e maior elevação da região posterior da língua^(26)^. Embora os achados desse estudo, após realização de canudo de alta resistência no ar, sugerem menor distintividade acústica entre as vogais, diminuindo a amplitude dos movimentos na região anterior da cavidade oral, eles podem indicar ampliação da orofaringe, pelo efeito do exercício^(26)^. Uma vez que, a elevação do complexo oromandibular resulta em ampliação do trato vocal próximo à glote, apresentando diminuição das frequências dos formantes^(3)^.

Por outro lado, estudo anterior^(27)^ investigou o CAR e concluiu que houve melhora na autopercepção de redução de esforço fonatório e menor fadiga vocal, apesar de não ter havido mudanças em outras medidas instrumentais.

Clinicamente, os exercícios investigados neste estudo podem ocasionar modificações a nível glótico e ressonantal, comprovados por meio da seleção de medidas acústicas, perceptivas e de autoavaliação^(13,19,21,22)^. No entanto, a investigação voltada para o espaço vocálico nos permite identificar que a forte comunicação da fonte glótica com as caixas de ressonâncias pode contatar os articuladores e provocar novos ajustes articulatórios, com possível influência na produção da vogal. Tal fato torna imprescindível a seleção de exercícios associados a fonte glótica e trato vocal, conjuntamente. É presumível também que o tempo de execução do exercício deve ser investigado por estudos futuros, e na clínica vocal, devem ser realizadas provas terapêuticas através do monitoramento multidimensional da voz.

Ao realizar a análise comparativa entre os grupos, observou-se que as diferenças entre os espaços vocálicos, principalmente no intervalo de F1 das vogais [a]-[i] permaneceram após os cinco minutos de realização do exercício com CAR, com valores maiores no MVS. É possível observar também que os participantes do MVS que executou o exercício de CAR apresentou maiores valores no intervalo de F1 das vogais [i]-[u] antes mesmo da execução desse exercício. Observou-se aumento da diferença no espaço vocálico entre mulheres do MNV e MVS após cinco minutos do exercício de VSL, com maior intervalo de F1 e F2 entre as vogais [i]-[u].

Sendo assim, a partir dos achados deste estudo pode-se verificar que há um maior intervalo no espaço vocálico de mulheres do MVS mesmo antes da execução do exercício, e que a sua execução parece potencializar essa diferença, principalmente para os exercícios de CAR e VSL. A redução do espaço vocálico também foi observada em estudos anteriores, incluindo indivíduos com paralisia unilateral de prega vocal^(9)^, com disartrias de diferentes etiologias^(4)^ e com disartria decorrentes da doença Parkinson^(5)^.

Possivelmente, mulheres com nódulos nas pregas vocais apresentam menor distintividade acústica em relação a mulheres vocalmente saudáveis, que parecem se beneficiar de maneira diferente do efeito dos exercícios vocais sobre o posicionamento dos articuladores. Pode-se inferir também o maior impacto (ou talvez um efeito mais imediato) dos exercícios vocais para melhora da clareza articulatória em indivíduos sem lesão laríngea em comparação com indivíduos com lesão, uma vez que, aumento nos intervalos de F1 e F2 entre as vogais altas e baixas influencia diretamente na maior clareza de fala^(28)^.

De modo geral, observa-se que a presença da vogal [i] em todos os intervalos dos espaços das vogais, trouxe algum resultado significativo na comparação entre o grupo MNV e MVS. Nesse sentido, pode-se inferir que na produção da vogal [i] a laringe configura-se em sua posição frontal mais alta^(3)^, com maior tensão e estabilização da laringe, com menor amplitude de vibração nas pregas vocais e altas taxas de pulsos glotais^(29)^. Portanto, a presença de nódulos vocais e o hiperfuncionamento laríngeo pode gerar interferência na produção do intervalo dessa vogal em relação às outras.

Com relação ao exercício de SA, não houve mudanças significativas em nenhum dos grupos. Em outro estudo, observou-se que o exercício de SA produziu efeito positivo imediato nos aspectos vocais e uma maior expressividade facial em pacientes com doença de Parkinson, principalmente após 15 minutos de realização^(20)^. Além disso, encontra-se que este exercício, associado com técnica de vibração sonorizada de língua e som nasal produziu melhora evidenciada nas medidas acústicas e no exame videoestroboscóbico da laringe em mulheres sem queixas vocais^(30)^. Dessa forma, pode-se pensar em duas possiblidades para esses achados: o tempo de execução do exercício, que não provocou modificações no espaço vocálico, ou a não associação à exercícios que envolvam a fonte glótica, os quais contribuiriam para o acoplamento fonte-filtro.

Portanto, este estudo apresentou evidências exploratórias a partir de três exercícios utilizados corriqueiramente na clínica vocal, com o intuito de ampliar o raciocínio crítico de fonoaudiólogos clínicos e pesquisadores acerca da relação entre a alteração laríngea e o posicionamento dos articuladores.

## LIMITAÇÕES E DIREÇÕES FUTURAS

Alguns fatores limitantes do estudo se referem ao tamanho da amostra presente em cada subgrupo para a realização dos exercícios vocais, o tempo de descanso entre as séries do exercício realizado, e a falta de outros parâmetros avaliativos com relação a produção e percepção vocal.

Para estudos posteriores, o interessante seria realizar a autopercepção vocal dos indivíduos presentes na amostra e um teste de inteligibilidade como uma medida de performance realizada por ouvintes, para descartar ou somar informações sobre esforço e fadiga muscular. Além de, aumentar o tamanho da amostra e estabelecer tempo de descanso entre as séries do exercício realizado.

## CONCLUSÃO

O exercício de VSL diminui o espaço vocálico em mulheres do MNV, com redução dos valores de F2 no intervalo das vogais [a]-[i]. O CAR reduz o espaço vocálico de mulheres do MVS, com diminuição dos valores de F1 nos intervalos [a]-[i] e [i]-[u], e diminuição dos valores de F2 nos intervalos [a]-[u] e [i]-[u]. No entanto, mulheres do MNV apresentam menor espaço vocálico em relação às mulheres do MVS, antes e após a realização do exercício de CAR. Houve redução do espaço vocálico das mulheres do MNV em relação ao MVS após o exercício de VSL. Na análise dos intervalos dos espaços das vogais, a vogal /i/ foi a que mais interferiu nos resultados. O exercício de AS não impactou de forma imediata no espaço vocálico.
